# Supplementary material for: Functional and Conformational Plasticity of an Animal Group 1 LEA Protein
Source: Biomolecules. 2022 Mar 10;12(3):425. doi: 10.3390/biom12030425 (PMC8946055; doi:10.3390/biom12030425)
Supplement: Supplementary file 1 [file biomolecules-12-00425-s001.zip › biomolecules-1578342-supplementary.pdf]

Supplemental Files
Functional and Conformational Plasticity of an Animal Group 1 LEA Protein
Brett Janis, Clinton Belott, Tyler Brockman, and Michael A. Menze

**Table S1:** Both *Af*LEA1 elution fractions display a tendency to elute a lower sodium concentration with increasing amounts of protein loaded.

| Elution Peak 1 | | Elution Peak 2 | |
|---|---|---|---|
| Elution Peak Volume (ml) | Peak Height (amu) | Elution Peak Volume (ml) | Peak Height (amu) |
| 30.52 | 19.14 | 34.69 | 45.45 |
| 30.49 | 16.98 | 34.69 | 44.90 |
| 30.37 | 21.63 | 34.56 | 47.88 |
| 30.33 | 19.44 | 34.47 | 50.19 |
| 30.09 | 23.92 | 34.45 | 50.10 |
| 30.09 | 23.92 | 34.45 | 50.10 |
| 30.04 | 23.92 | 34.44 | 50.09 |
| 29.75 | 24.16 | 34.02 | 59.06 |

**Figure S1**

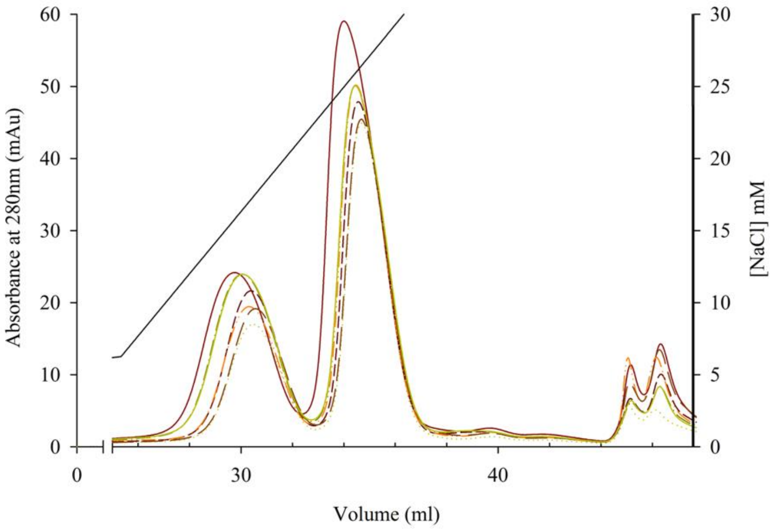


Figure S1: The elution maxima (retention time) of *Af*LEA1 tends to correlate with the amount of loaded protein (peak height). The concentration of *Af*LEA1 used on the column negatively correlates with the NaCl concentration required to reach peak elution. Both major elution fractions display a shift of the elution maximum to the left with increasing loading of *Af*LEA1 onto the column (c.f., Supp. Tab. 1).

**Figure S2**

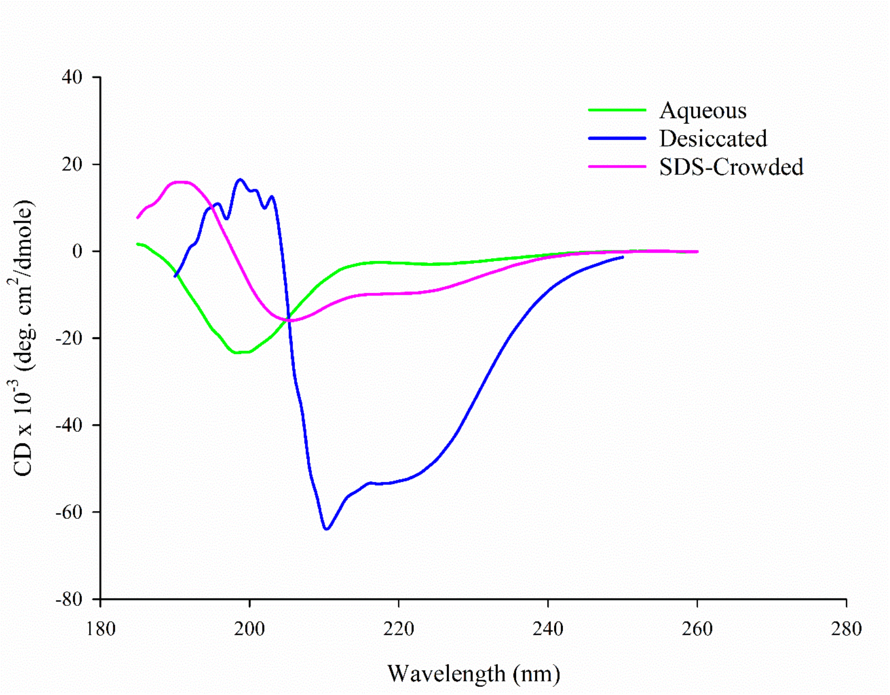


Figure S2: The CD spectra of *Af*LEA1 measured in solution (green), in the desiccated state (blue), and when crowded via 2% v/v SDS (magenta). Relevant buffer samples were subtracted from the spectra for hydrated protein samples, whereas an empty cuvette was measured as a blank for the desiccated sample.

**Figure S3**

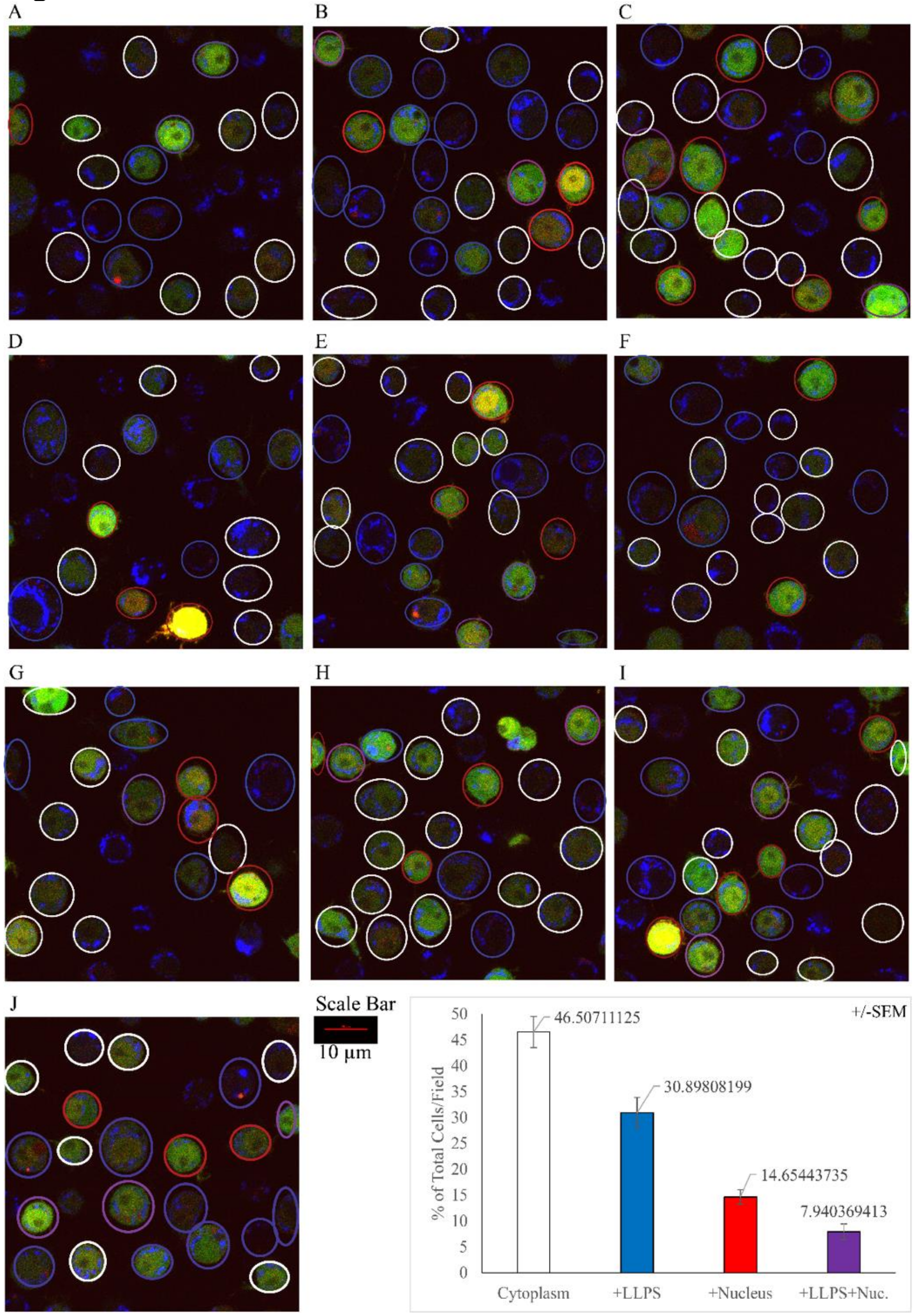


Figure S3: Relative frequencies of unique morphologies observed in Kc167 *Drosophila melanogaster* cells ectopically expressing *Af*LEA1-mCherry (A-J). *Af*LEA1-mCherry was found to localize to the cytosol in all cells, with ~46% of cells (white circles) showing no other localizations. In addition to remaining in the cytosol, *Af*LEA1-mCherry was observed in ~30% of cells (blue circles) to undergo a liquid-liquid phase separation (LLPS) without entering the nucleus. *Af*LEA1-mCherry was also observed in ~15% of cells (red circles) not to show any visible

LLPS, and instead, the protein accumulated in the nucleus. Lastly, ~8% of Kc167 cells (purple circles) showed both LLPS of *Af*LEA1-mCherry and accumulation of the protein in the nucleus. A total of 204 cells were qualitatively assessed. Cells with no detectible *Af*LEA1-mCherry fluorescence were not included in the evaluation. Fluorescence is relative among images.
